# Biological shaping as a conservative alternative for crown lengthening: A review

**DOI:** 10.1002/cre2.873

**Published:** 2024-03-20

**Authors:** Abdusalam Alrmali, Daniel Melker, Janet Zalucha, Hom‐Lay Wang

**Affiliations:** ^1^ Department of Periodontics and Oral Medicine University of Michigan School of Dentistry Ann Arbor Michigan USA; ^2^ Department of Oral Medicine, Oral Pathology, Oral and Maxillofacial Surgery University of Tripoli School of Dentistry Tripoli Libya; ^3^ Periodontist private practice limited to Periodontics Clearwater Florida USA

**Keywords:** biological width, furcation defects/surgery, prosthodontic/methods*, tooth preparation

## Abstract

**Objectives:**

The perio‐restorative approach to maintaining supracrestal tissue attachment (STA; formerly known as biologic width) is a fundamental goal in modern dentistry. This article aims to review the clinical impact of biologic shaping (BS) as an innovative alternative to traditional crown lengthening procedures, reflecting over two decades of clinical experience.

**Material and Methods:**

As a review paper, it is crucial to highlight that BS stands as a unique approach designed to optimize STA while emphasizing minimal to no removal of supporting bone. The review spans over two decades, consistently demonstrating clinical efficacy and predictability. Remarkably, BS focuses on addressing issues such as root concavities, developmental grooves, irregularities, furcation lips, and CEJ offering a remarkable level of clinical precision.

**Results:**

The reviewed literature underscores that BS has consistently achieved substantial clinical success in fulfilling its objectives. This method presents a biologically sound alternative to traditional crown lengthening, placing a strong emphasis on the preservation of essential bone tissue and the establishment of durable STA.

**Conclusions:**

The results suggest that BS is a logical and biologically driven approach for maintaining STA, making it a promising alternative to traditional crown lengthening. The method offers a predictable and reproducible way to preserve bone tissue while achieving durable STA. This innovation holds great promise in the field of periodontal and restorative dentistry.

## 1 INTRODUCTION

Crown lengthening is a well‐established surgical procedure in periodontal practice, commonly employed to address restorative and esthetic concerns by increasing the clinical crown height (Bennani et al., 2017; Marzadori et al., 2018). In conventional crown lengthening (CCL), the amount of bone removal (typically 3–4 mm) is determined using existing crown margins or the cementoenamel junction (CEJ) of an unrestored tooth as a reference point to create space for supracrestal tissue attachment (STA), previously referred to as biologic width (Melker & Richardson, 2001). However, a significant limitation of CCL lies in the removal of a substantial amount of supporting bone through ostectomy and osteoplasty. This procedure can lead to an unfavorable crown‐root ratio, expose more root anatomy, particularly at the furcation area, and pose challenges for future dental plaque control. Such outcomes may create an environment that is biologically incompatible and challenging to maintain (Rosenstiel et al., 2022).

In contrast, the biologic shaping (BS) technique, introduced over two decades ago by Melker, offers an alternative and conservative approach to traditional crown lengthening (Melker & Richardson, 2001; Strupp WC & Melker, 2003). BS primarily focuses on the root surface rather than bone destruction, moving the existing margins away from the bone and only removing bone when absolutely necessary (Melker & Richardson, 2001). This innovative technique effectively eliminates root concavities, irregularities, furcation lips, and CEJ issues. Over more than two decades, BS has consistently demonstrated clinical success by establishing STA with minimal or no supporting bone removal (Tucker et al., 2012). Consequently, the restorative dentist can consistently place a fine chamfer margin of 0.3–0.5 mm in a supragingival position, avoiding STA invasion, which can lead to inflammation and host response activation, potentially resulting in the destruction of periodontal supporting tissues (Melker & Richardson, 2001, Melker, 2009). The aim of this article is to revisit the BS technique after two decades of clinical innovation and assess its impact as a conservative alternative to CCL, with a particular focus on its benefits and long‐term clinical success.

## 2 TECHNIQUE

The original study protocol underwent rigorous ethical scrutiny and received approval from the Ethics Committee, specifically for data collection and the sharing of photos. Patients who participated in the original study provided explicit written consent. As an essential aspect of ensuring patient privacy and confidentiality, specific patient files and data were meticulously safeguarded. It is worth highlighting that the original analysis adhered to the ethical principles outlined in the World Medical Association Declaration of Helsinki, ensuring that the study was conducted with full regard for the rights and well‐being of the participants.

### 2.1 BS for anterior and premolar teeth

**Figure 1:**
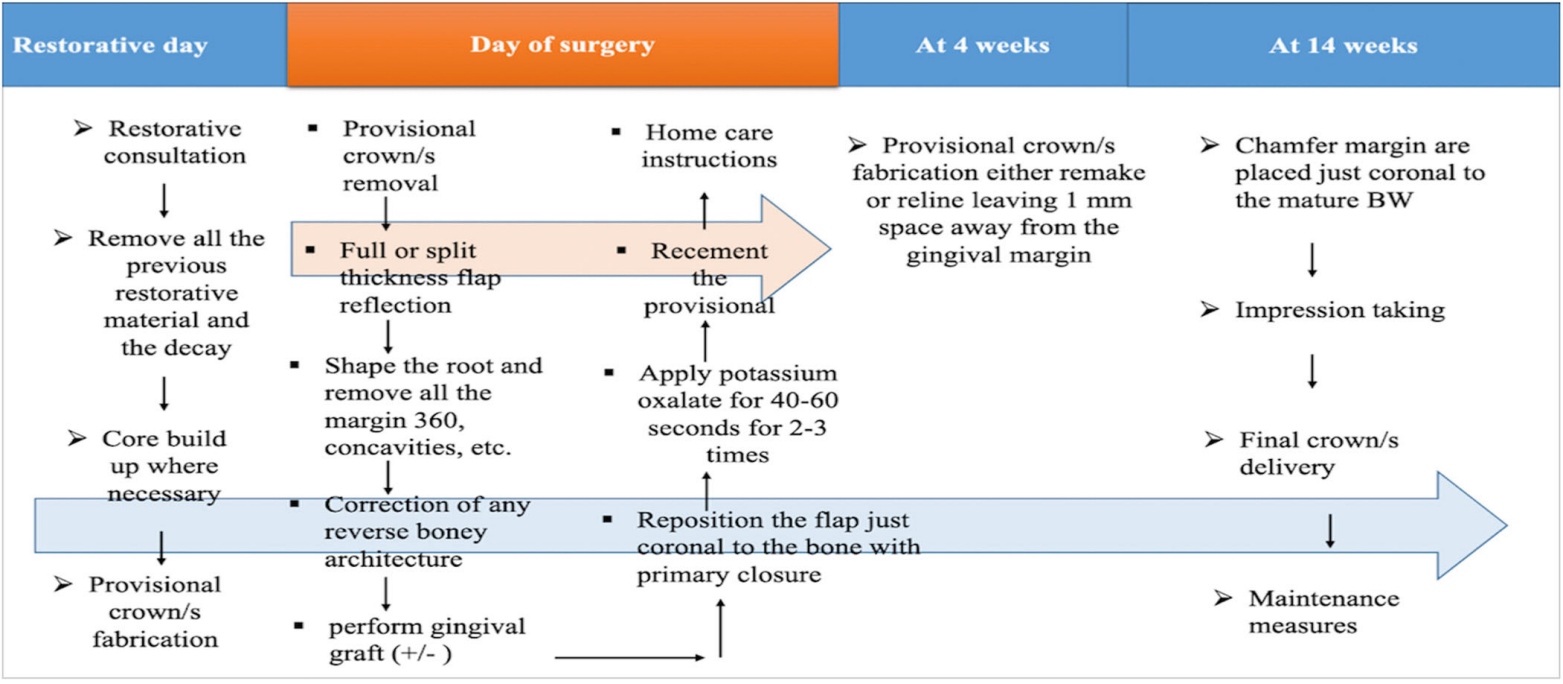
The clinical steps for biological shaping procedure.

**Figure 2:**
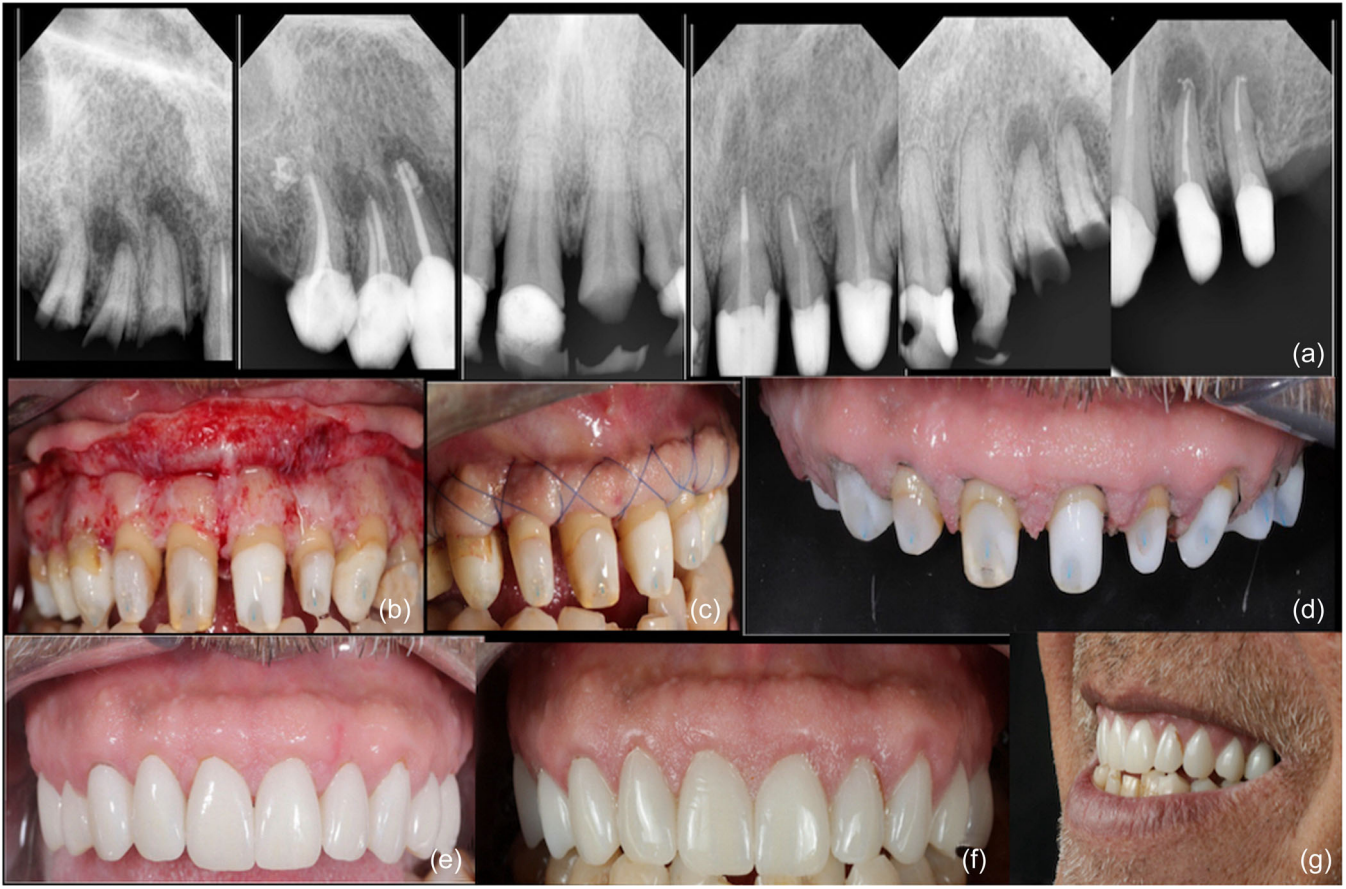
Case with anterior and premolar teeth requiring new restorations, root canal treatment, and crowns. (a) Pre‐and postoperative radiographs were taken to assess the treatment outcome. (b) Incisions were made using a #15 scalpel blade, and a full‐thickness flap was elevated until the mucogingival junction. (c) Frontal view shows the use of continuous periosteal sutures to hold the flap in an apical direction just coronal to the bone. (d) Final crown margins are now supragingival to the previous crown margin. (e) Clinical images for the E‐Max restorations (Multilink; Ivoclar AG) were taken at a 1‐year follow‐up to assess the long‐term outcome of the treatment. (f, g) Intraoral and extraoral images of the patient's smile after 3 years follow‐up, showcasing the successful outcome of the case. The prosthetic workflow was completed by Moneum Shembesh and Elhussein Gnao.

**Figure 3:**
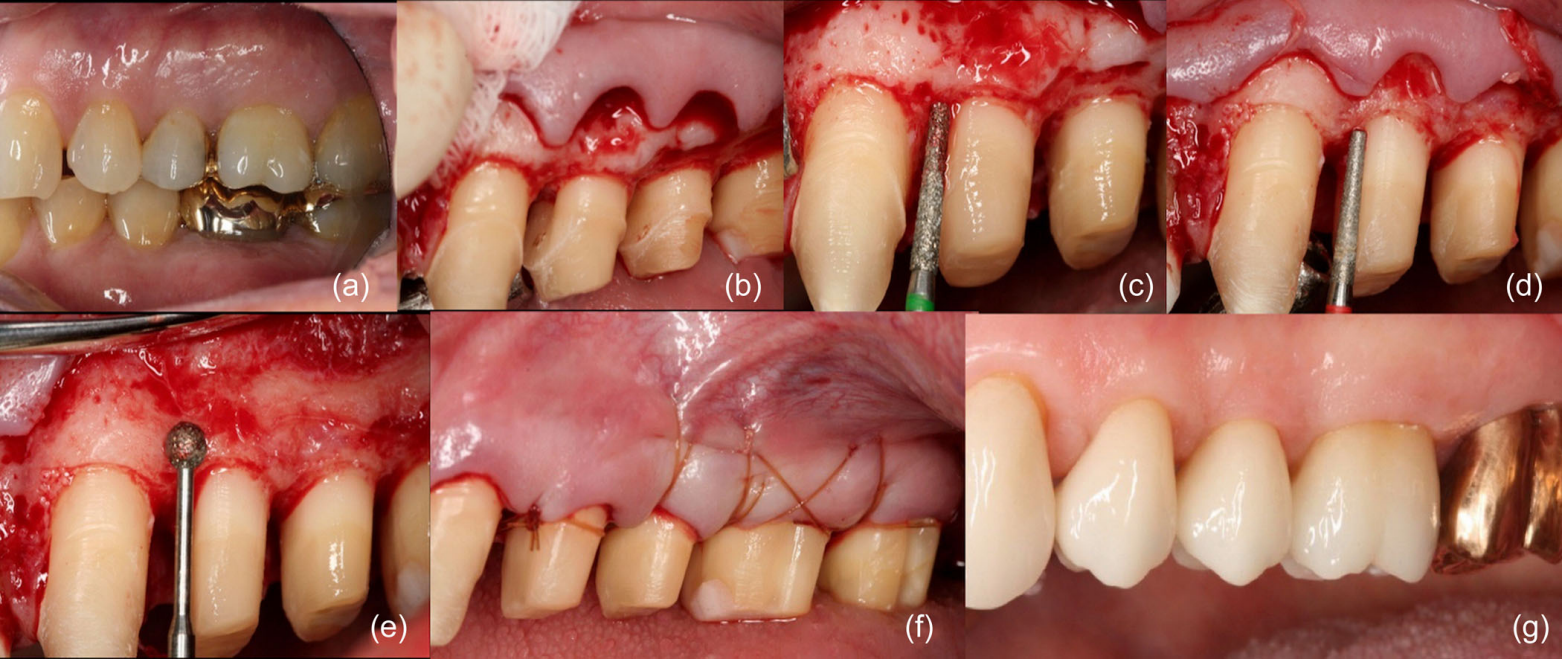
(a) Initial image revealing occlusal problems associated with temporomandibular disorder, leading to a treatment plan involving previsualizations of maxillary teeth for occlusal correction. (b) Incisions were meticulously made, elevating a full‐thickness flap while preventing perforation due to underlying exostosis, with a split‐thickness flap created for suturing to periosteum upon closure. The root surface was smoothed, and granulation tissue cleared. (c, d) Employing coarse and superfine burs achieved a polished root surface. (e) Creation of a parabolic architecture and bone contouring using a diamond round bur confirmed complete furcation removal. (f) Suturing utilized a Castro‐Viejo and 5‐0 chromic gut material for primary coverage. (g) Placement of E‐Max restorations (Multilink; Ivoclar AG) with full‐coverage gold, completing the case spanning over 5 years, and finalized by Howard Chasolen.

Figure 1 shows the clinical steps for the BS procedure. Figures 2 and 3 show the step‐by‐step process of BS for anterior and premolar teeth.

BS begins with the removal of any existing carious lesions, restorations, crowns, or bridges, followed by placing a solid bonded resin foundation restoration and/or preparing a feather‐edge margin on the tooth for an interim crown that should be kept 0.5–1 mm away from the gingival margin (Melker & Richardson, 2001; Melker et al., 2020; Strupp WC & Melker, 2003, Melker, 2009). On the day of the surgery, the surgeon removes the provisional crown and reflects a full‐thickness flap in most cases to perform osseous contouring 360° on the buccal and lingual sides. Soft tissue, bone, and root surfaces are then treated accordingly by removing all granulation tissues, bone exostosis, furcation lips, developmental grooves, root concavity, and enamel projections that may compromise future tissue adaptation and cleansability (Melker & Richardson, 2001; Melker et al., 2020). Table 1 demonstrates a comparison of functional crown lengthening versus BS.

**Table 1:** summarizes the comparison between functional crown lengthening (FCL) versus biologic shaping (BS).

Main factors to consider	FCL	BS
Goal	Increase the amount of tooth height by removing 3–4 mm from the reference point	Reshaping the existing tooth surfaces in combination with preserving alveolar bone
Reference point	Margin of the old preparation or restoration margin	Create the width needed for the restoration to be biologically acceptable
Crown/root ratio	Increased	Maintained
Osseous surgery	Bone is removed from the adjacent teeth too	Bone is slightly removed from the tooth in question and maintained
Root reshaping	Never	Core of BS
Restorative work	Intraoperative or postoperative	Preoperative “if we can core it then we can save it”
Provisional crown/s	Not often, postsurgical	Mandatory presurgically
Furcation area	Expose more furcation area by removing more bone	Preserve the bone at furcation and work on the root line angles
Wound dressing	Often required	Not necessary
Keratinized gingiva	Same or decrease	Increase
Cleansability of subgingival root portion	Worse	Better maintained

This established protocol demonstrates its versatility, as it can be applied to any tooth requiring treatment. The underlying rationale for adopting the BS technique is grounded in several significant biological factors, ensuring its long‐term stability and clinical efficacy.

1. *Expanded indications*: The BS technique extends beyond the conventional indications of functional crown lengthening, offering a broader spectrum of applicability.
2. Minimal ostectomy: Unlike traditional approaches that necessitate ostectomy, BS operates on the principle of modifying the root surface rather than the bone, thereby minimizing bone removal.
3. *Preservation of biologic width*: The technique facilitates the establishment of supragingival or intrasulcular margins, effectively preserving the biologic width and avoiding complications associated with its violation.
4. *Elimination of developmental grooves*: BS successfully eradicates developmental grooves that can serve as potential sites for bacterial colonization and subsequent periodontal complications.
5. *Coronal relocation of margins*: By relocating previous subgingival restorative margins more coronally, BS contributes to enhanced clinical outcomes and facilitates improved oral hygiene maintenance.
6. *Furcation anatomy modification*: The technique's ability to reduce or eliminate furcation anatomy enables optimal margin placement on smooth, parallel roots, streamlining restorative procedures.
7. *Gentle impression techniques*: BS allows for the adoption of supragingival or intracrevicular impression techniques, minimizing trauma to connective tissue fibers during the impression‐taking process.

The BS technique thus emerges as a comprehensive and biologically driven approach that addresses a range of clinical considerations, promoting both the stability of treatment outcomes and the preservation of the tooth's natural structure. The exposed dentin surfaces are treated with desensitizing agents, after which the interim restorations are modified to ensure that all margins are at least 1 mm supragingival. The restorative dentist relines the interim restorations 4 weeks after surgery, and the newly relined interim restoration is also placed 1–2 mm supragingival. The surgical site is allowed to reestablish a healthy STA, and at approximately 4 months, light chamfer margins are placed slightly supragingivally, after which the restorations are completed. Figure 1 summarizes the clinical steps for the BS procedure. In addition, the following cases are examples of BS for different teeth (Figure 2a–h, BS for anterior teeth; Figure 3a–g, BS for premolar teeth).

### 2.2 BS for molar teeth

**Figure 4:**
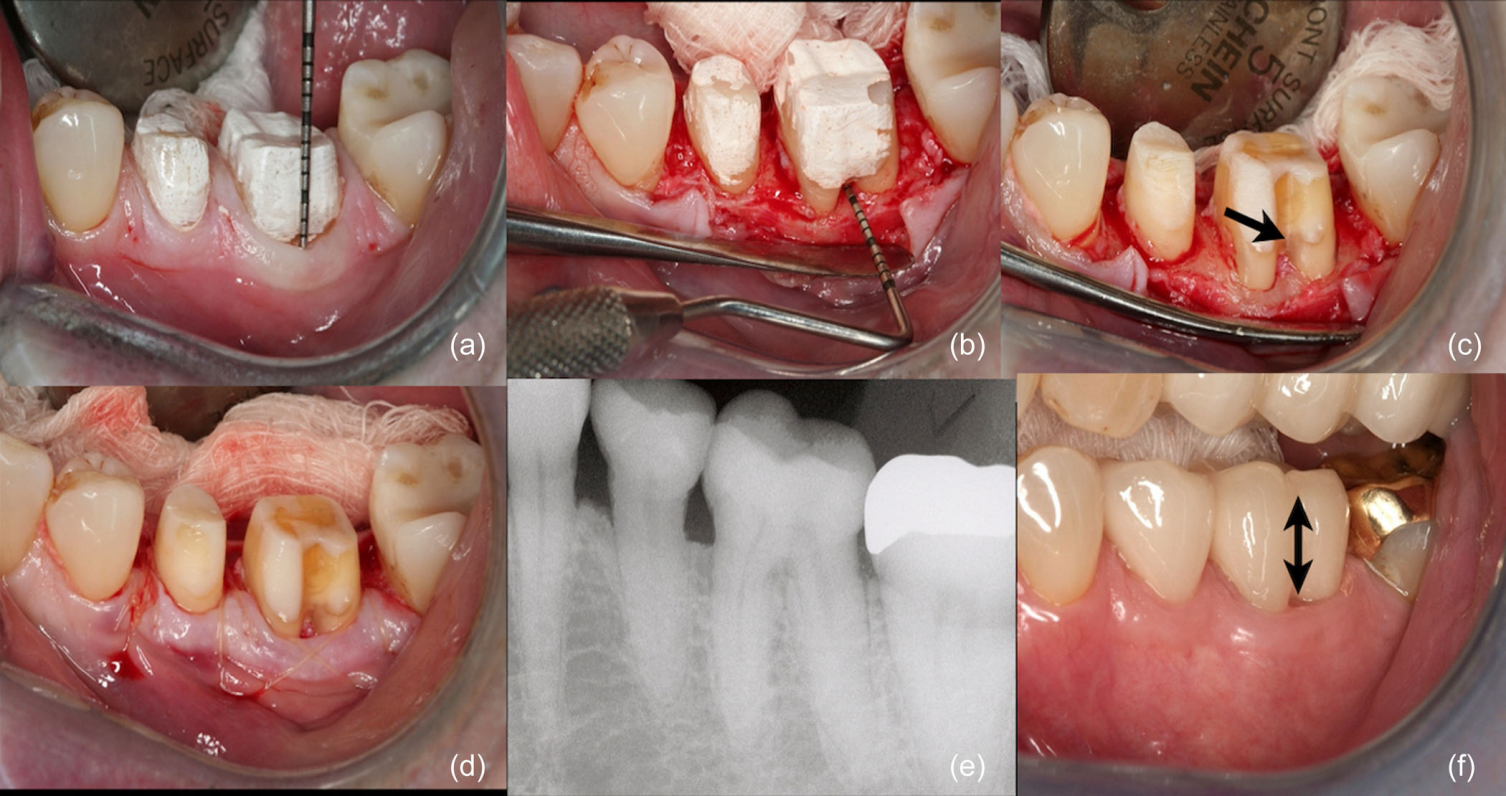
Referral was made for addressing STA (subgingival tooth structure approximation) invasion, following prior treatment involving restoration and decay removal, core placement, and acrylic‐based provisional restoration (polymethyl methacrylate, PMMA, Multilink; Ivoclar AG). (a) Provisionals were taken off, with Durelon cement (zinc oxide‐eugenol cement, 3 M) retained for antimicrobial protection. (b) A full‐thickness flap was elevated, and a partial‐thickness dissection performed apical to the mucogingival junction. (c) Biologic shaping yielded a seamlessly smooth tooth structure, spanning bone to occlusal surfaces, without any margin. Precise furcation bone positioning was crucial, as furcation removal induced bone coronal shift (black arrow). (d) Suturing positioned just coronal to the bone ensured primary closure and reduced postoperative discomfort. (e) Final restorations were radiographically confirmed and completed for stability and functionality spanning over 15 years (black arrow). Restorations executed by William Strupp Jr.

**Figure 5:**
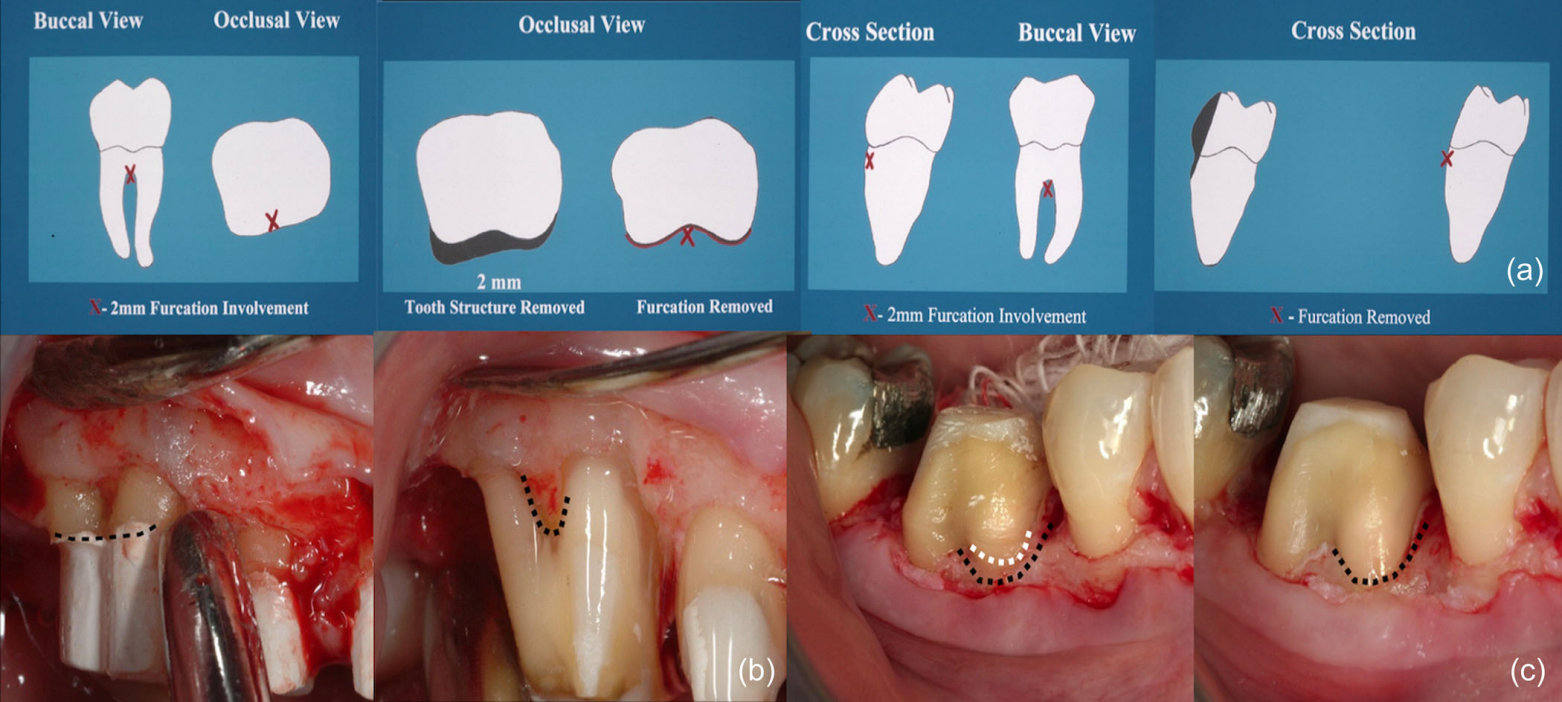
(a) The letter “x” serves to pinpoint the furcation's location in buccal and occlusal views. (b) Shaded regions indicate areas, especially near the furcation, where tooth structure should be removed to prevent the formation of challenging‐to‐clean deep grooves. The second image's “x” indicates furcation absence within the tooth structure. These images portray the furcation's position in a cross‐sectional view, with shading denoting necessary tooth structure removal for furcation elimination. Maintaining the absence of height of contour in the final restoration is vital. In the right photograph, the “x” is now beyond the tooth surface, signaling furcation eradication. The barreled‐in furcation should extend to the occlusal surface to avert non‐cleanable zones. (b, c) Illustrating upper and lower furcations, these images underscore the need for the existing margin to approximate the furcation (black and white dots). Biologic shaping yields complete smoothness from bone to occlusal surfaces (black dots).

Figures 4 and 5 show the clinical steps for the BS procedure for molar teeth.

The intricate nature of furcation lesions poses distinctive challenges for both periodontists and restorative dentists. In the context of molars necessitating periodontal‐restorative intervention, it becomes imperative to explore alternative approaches to CCL procedures. This consideration gains significance due to the potential implications of furcation involvement, where the invasion of supracrestal fibers and the widening of the furcation can occur. This scenario arises from the dynamics of bone movement within the furcation area when attempts are made to extend the crown in this region, the furcation bone typically shifts coronally. Consequently, the conventional approach of creating space for STA becomes impractical, as it would involve compromising the furcation bone extensively, leading to potential long‐term tooth complications. The more substantial the bone removal within the furcation, the higher the risk of future maintenance‐related issues. Thus, the preservation of adequate bone support, particularly in the furcation, stands as a critical consideration (Melker et al., 2020).

Considering this, a more prudent strategy is advocated; one that commences by eliminating the restorative margin or CEJ, followed by meticulous shaping of the tooth structures surrounding the furcation. This preparatory phase (depicted in Figures 4a–f and 5a–c) lays the groundwork for informed decision‐making regarding the necessity of ostectomy. Crucially, this approach is universally applicable to teeth with both shallow and deep furcations, and its advantages are particularly pronounced in cases requiring full‐coverage restorations. The skillful execution of this approach leads to the eventual elimination of the furcation defect. Confirmation of success is marked by the ability to insert a periodontal probe at the furcation bone level, allowing for smooth movement without encountering any coronal undercuts. Ultimately, the restoration of the tooth facilitates improved hygiene practices for both patients and hygienists, thereby contributing to long‐term oral health.

## 3 DISCUSSION

The functional (restorative) crown lengthening procedure is indicated to facilitate restorations in teeth with compromised crown structure or to avoid violating the attachment apparatus (STA) by exposing more tooth structure supragingivally (Melker et al., 2020, Melker, 2009). However, if the restoration margin is extended subgingivally into the STA zone, bacterial infiltration with subsequent gingival inflammation can develop within the attachment apparatus (Waerhaug, 1978). The STA, or supra‐bony components of the root, are defined as the vertical dimension of the dento‐gingival complex, which includes the gingival sulcus, junctional epithelium, and connective tissue attachment (Gargiulo et al., 1961). Although most clinicians are familiar with the concept of STA, some confusion exists regarding its relevance to clinical procedures (Ingber et al., 1977).

According to a human study that analyzed 287 individual teeth from 30 autopsy specimens, a definite measurement was established between the alveolar crest, connective tissue attachment, and epithelial attachment (Gargiulo et al., 1961). They reported specific extents for each part as follows: a sulcus depth of 0.69 mm; an epithelial attachment of 0.97 mm; and a connective tissue attachment of 1.07 mm. Based on their work, the STA is commonly stated to be the sum of the epithelial and connective tissue measurements, namely a value of 2.04 mm (Gargiulo et al., 1961). However, the measurements are not fixed and can vary even around the same tooth. Significant clinical variations of these dimensions were observed, particularly of the epithelial attachment, which ranged from 1.0 to 9.0 mm. Another systematic review found similar mean values of biologic width (2.15–2.30 mm), although considerable intra‐ and interindividual variances were reported (subject sample range: 0.20–6.73 mm) (Gargiulo et al., 1961; Marzadori et al., 2018).

It is commonly believed that a minimum distance of 3 mm should be maintained between the restorative margin and the alveolar bone to avoid violating the STA and its deleterious effects (Padbury et al., 2003). However, this approach will destroy the bone that serves as a critical supporting structure for the tooth. A more biologically sound approach would be to move the finish line away from the bone and thicken the 1 mm connective tissue barrier, which would provide better biological protection. This is the core principle of the BS technique. It has been suggested that the only tissue that can prevent bacterial infiltration is the 1 mm connective tissue that is measured from the bone crest, and adding an abundance of connective tissue is key to achieving a successful perio‐restorative interface. Furthermore, it has been suggested that the margin could be placed between 0.3 and 0.5 mm below the gingival tissue crest for even better results (Kois, 1994; Nugala et al., 2012).

Concurrently, if the restoration margin is extended subgingivally into the biological width zone, bacterial infiltration with subsequent gingival inflammation will develop within the attachment apparatus (Waerhaug, 1978). The presence of chronic inflammation will affect adult cells and their interaction with the periodontal healing potential and must be addressed when planning shaping techniques. In cases where infections are present, appropriate management strategies should be implemented through the removal of the old prosthesis and/or performing phase one periodontal therapy to ensure the best possible clinical outcomes. Nevertheless, subgingival margins should be considered a compromise and better be avoided unless in high esthetic cases or discolored abutment (Bennani et al., 2017; Lang & Löe, 1972; Schätzle et al., 2001). In such cases, the margin should be placed from 0.3 to 0.5 mm subgingival to avoid the risk of violating the CTA (Kois, 1996). However, an important factor that should be taken into consideration is the presence of enough connective tissue thickness. If there is insufficient tissue thickness, tissue volume and/or keratinization may need to be increased when placing the restorative margin subgingivally (Stetler & Bissada, 1987). For more than 20 years, BS has been used as an alternative to crown lengthening in such cases, with the main focus on establishing the finish line but not destroying bone. BS stands as an invaluable therapeutic technique with a versatile range of applications. One of its key strengths lies in its efficacy in reshaping fractured teeth with borders that typically extend to or just under the bone crest. In cases of dental fractures, where the traditional approach might necessitate excessive bone removal to accommodate restorative interventions, BS offers a more conservative alternative. By focusing on the root surface and adapting the finish line to minimize or eliminate bone removal, this technique allows for the preservation of vital bone structure, thereby promoting the long‐term stability of the treated tooth.

Moreover, BS presents an innovative solution for scenarios where interproximal spaces are limited due to the close proximity of adjacent roots. Conventional approaches might involve more aggressive interventions, but BS offers a more nuanced strategy. Through careful manipulation of the root surface and bone contours, this technique provides the means to create additional space between the roots without compromising the integrity of the surrounding structures. This application of BS holds promise in addressing challenges related to interproximal spaces, contributing to enhanced treatment outcomes and patient satisfaction (Kois, 1996; Melker et al., 2020). Therefore, the ultimate goal is to open a flap to access the root surface as well as for osseous recontouring. This involves removing bone ledges and thick bone to create a parabolic architecture that mimics the soft tissue so that when the flap is readapted, it can heal optimally. Thick bony exostosis and bone ledges can create an environment that is unhealthy for the surrounding tissues (Carnevale & Kaldahl, 2000; Chiu et al., 2022). The limitations of BS are minimal. It guarantees significant improvement compared with full crown lengthening, but it requires close coordination between highly skilled restorative and periodontist team members. As dentists, our primary goal should be to keep the natural tooth for as long as possible, and BS is one of the options to achieve this goal.

## 4 CONCLUSIONS

BS is a technique that preserves the bone while establishing the necessary STA for the restoration of teeth. It helps improve hygiene measures for both patients and dental professionals and creates a biocompatible environment required for long‐term success. When performed correctly, BS can enhance the short‐ and long‐term prognosis of teeth.

## AUTHOR CONTRIBUTIONS

Conception and design of the study; performed the surgical procedures, initial and final drafting of the work: Abdusalam Alrmali and Daniel Melker. Initial and final drafting of the manuscript: Janet Zalucha. Design of the study; critical review of the draft and contribution to the writing of the manuscript: Hom‐Lay Wang. All authors gave their final approval of the version to be published and are accountable for the accuracy or integrity of the work.

## CONFLICT OF INTEREST STATEMENT

The authors declare no conflict of interest.

## Data Availability

The data that support the findings of this study are available from the corresponding author upon reasonable request.
